# Supplementary material for: One Hundred Years of Coat Colour Influences on Genetic Diversity in the Process of Development of a Composite Horse Breed
Source: Vet Sci. 2022 Feb 6;9(2):68. doi: 10.3390/vetsci9020068 (PMC8879117; doi:10.3390/vetsci9020068)
Supplement: Supplementary file 1 [file vetsci-09-00068-s001.zip › Table S1.pdf]

Table S1. Genetic background of coat colour phenotypes described in Spanish Purebred, Arabian Purebred and Hispano-Arabian horse breeds.

Locus/Most Plausible Candidate Gene	Chromosome	Nomenclature	Alleles	Allelic combinations	Effect of combined pairs of alleles			Phenotype	Gene ID	Observations
					Skin	*Hair*	*Eyes*			
Melanocortin 1 receptor *(MC1R)*	3	Extension	E<br>e<br>e^a	EE, Ee, or Ee^a	Black	Black	Dark	Black, seal brown, or bay	100136907	
				ee, ee^a, or e^ae^a	Black	Red	Dark	Chestnut/Sorrel		
Agouti-signaling protein *(ASIP)*	22	Agouti	A<br>a	AA or Aa	Black	Black in points/Red	Dark	Bay or Chestnut/Sorrel	100054335	
				aa	Black	Black	Dark	Black or Chestnut/Sorrel		
Solute Carrier Family 45 Member 2/Membrane-associated transporter protein *(MATP/ SLC45A2)*	21	Cream/Pearl (also called “apricot” or “Barlink factor”)	Cr<br>prl<br>C, Prl, or n	C^{Cr}/C^{Cr}	Pale	Red	Blue	Cremello, perlino/pearl, or smoky cream (double dilute cream)	100068242	The dominant cream gene will activate the pearl phenotype if the two genes are present. The C^{Cr} allele does not occur in Arabian horses. Therefore, there are no cream arab horses, and also no palomino, buckskin or smoky black horses either.
				C^{prl}/C^{prl}	Pale	Red	Dark/Blue	Double Pearl, Isabelline/Isabella		
				C^{Cr}/C	Dark/Pale which darkens with age	Red	Dark/Dark blue-gray	Palomino, buckskin, or smoky black (single dilute cream)		
				C^{Cr}/C^{prl}	Pale	Red	Blue/Green	Pseudo-double dilute. Horses positive for both cream and pearl will exhibit a double-dilute phenotype, again with a highly pale coat color similar to that of horses that are homozygous for the cream gene. Known as cream pearl in the Purebred Spanish horse breed.		
				C/C^{prl}	Black or Pale	Black or Red	Dark or Pale	No visual change to carrying horse, except for in the additional presence of the cream dilution gene (single dilute pearl)		
				C/C	Black	Black or Red	Dark	Normal, undiluted.		
T-box transcription factor *(TBX3)*	8	Dun/Wildtype	D<br>ND1<br>ND2 or d	DD, D/ND1, or Dd	Black	Red	Dark	Pinkish-red, yellow-red, yellow or mouse grey, grullo (blue dun) with at least one primitive marking including dorsal stripe, shoulder stripe and leg barring.	100056019	
				ND1/ND1	Black	Black or Red	Dark	Horse is not diluted but has a darker dorsal stripe and may have other primitive markings		
				ND1/d	Black	Black or Red	Dark	Horse is not diluted, but has faint primitive markings.		
				dd	Black	Black or Red	Dark	Undiluted with no primitive markings.		
Syntaxin 17 *(STX17)*	25	Grey	G<br>g	GG or Gg	Black	Black and/or Red and White	Dark	Progressive silvering with age to white or flea-bitten, but is born a non-gray color. White to dark grey depending on age and the proportion of white hairs in the coat.	100054797	
				gg	Black or Pale	Black and/or Red	Dark	Does not gray with aging		
Endothelin B receptor *(EDNRB)*	17	Frame Overo/Lethal white syndrome	O or Fr<br>o or fr	Oo	Black and Pale	Black, Red and White	Dark or Blue	Frame Overo pattern - Pinto horse pattern that forms a solid frame around white spotting. White is usually horizontal in orientation with jagged edges, color crosses the back and legs, face is often white.	100033875	The Overo "O" allele is different from overo as a color pattern classification in those registries which also include the sabino genes under the heading "overo."
				oo	Black or Pale	Black or Red	Dark	No overo pattern present.		
				OO	Pale	White	Blue	Homozygous overo is lethal white syndrome, characterized by an incomplete colon and the inability to defecate, which leads to death or humane euthanization within 72 hours of birth.		
Inversion starting about 100k bp downstream of proto-oncogene, c-KIT receptor tyrosine kinase (c-KIT)	3	Tobiano	TO<br>to	TOTO or TOto	Black and pale-skinned patches	Black, Red and White patches	Usually Dark, but one or both may be Blue or partially Blue	Tobiano, a form of pinto patterning. Produces regular and distinct ovals or rounded patterns of white and color with a somewhat vertical orientation. White extends across the back, down the legs, but face and tail are usually dark.	100097704	
				toto	Black or Pale	Black or Red	Dark	No tobiano pattern present.		
c-KIT or CD117 (cluster of differentiation 117)	3	White, Sabino	W1, W2, W3, W4, W9, W10, W11, W13, W14, W17, W23, W24, W25, W27, SB1 or n	W/W	Pale	Entirely or almost entirely white	Blue	Thought to be lethal. Embryo reabsorbed or fetus dies *in uterus*.	100097704	Complex. Sabino is registered as overo by some registries, but is not frame overo and does not cause overo lethal white syndrome.
				W/n, W5/W20, W20/W22, or SB1/SB1 (Maximum Sabino)	Pink	White	Dark	Hair coat is white from birth. There may be some patches of color, which may fade to white as the horse grows older.		
				SB1/n (Classic sabino)	Pink	White	Dark or Blue (But not linked to SB1)	Assorted pinto or roan-like markings. abundant white on the legs, belly spots or body spots that can be flecked or roaned, chin spots, or white on the face extending past the eyes.		
				n/n	Black or Pale	Black and/or Red	Dark	Horse is fully pigmented.		
Near or at *c-KIT*	3	Roan	Rn<br>rn	RnRn or Rnrn	Black or Pale	Black or Red with interspersed White hairs	Dark	Even mixture of interspersed white and colored hairs in the coat which are more scattered or absent on the head, mane, tail, and lower legs. The unaffected color on the legs often forms a sharp, inverted "V" above the knee and hock, not seen in other roan-like coat patterns. The non-white underlying coat may be any color, as determined by unrelated genetic factors. Black roans are referred to as blue roans, bay roans as red roans, and chestnut roans as strawberry roans, but there are also references to purple roans, lilac roans, and honey roans. Further, a “red roan” could have either bay or chestnut as the underlying color, while some dark bay roans were called blue roan or purple roan.	100097704	Gene not present in Arabians, although coat officially appears in registries. Roan in Arabians is probably associated with perhaps sabino. Unlike with the traditional Roan gene, in Arabians a roan offspring can be produced by non-roan parents.
				rnrn	Black	Black	Dark	No roan pattern.		
Melanocyte inducing	16	Splashed white	SW1<br>SW3<br>n	SW1/SW1	Black and Pale under White markings	Black or Red with White markings	Dark/Blue	Classic splashed white.		The first record for this pattern dates back to

transcription factor *(MITF)*				SW1/n	Black and Pale under White markings	Black or Red with White markings	Dark/Blue	White markings on head and legs.	1000 3391 8	2006, when Splashed White mutation occurred spontaneously in a PRE bay filly with a white bonnet, blue eyes, and white stockings was born to two solid bay parents. DNA parentage testing confirmed that the filly was truly the daughter of her proposed parents and she was registered as Unica LXXX and reproted to have a bay coat (her solid colour underneath).
				SW3/SW3	Letal	Letal	Letal	May be embryonic lethal		
				SW3/n	Black and Pale under White markings	Black or Red with White markings	Dark/Blue	Splashed white.		
				n/n	Black and Pale	Black or Red	Dark	No splashed White.		
Paired box gene 3 *(PAX3)*	6		SW2<br>SW4<br>n	SW2/SW2	Black and Pale under White markings	Black or Red with White markings	Dark/Blue	Classic splashed white.	1000 5651 7	
				SW2/n	Black and Pale under White markings	Black or Red with White markings	Dark/Blue	Splashed white, but usually not as loud as a classic splash		
				SW4/SW4	Letal	Letal	Letal	Might be lethal.		
				SW4/n	Black and Pale under White markings	Black or Red with White markings	Dark/Blue	Splashed white or broad blaze.		
				n/n	Black and Pale	Black or Red	Dark	No splashed White.		

Accessed from Sponenberg and Bellone (2017)
