# Supplementary material for: One Hundred Years of Coat Colour Influences on Genetic Diversity in the Process of Development of a Composite Horse Breed
Source: Vet Sci. 2022 Feb 6;9(2):68. doi: 10.3390/vetsci9020068 (PMC8879117; doi:10.3390/vetsci9020068)
Supplement: Supplementary file 1 [file vetsci-09-00068-s001.zip › Table S2.pdf]

Table S2. Summary of the results derived from offspring analyses and stallion/ratios across coat colour subgroups in historic and current population sets.

Population Set / Parameter	Population set	Grey	Bay	Chestnut/Sorrel	Black	Overo	Roan	Dun	White	Isabelline	Cremello	Pearl	Palomino	Smokey Cream
Stallions %	Historical	47.81	47.82	46.17	48.27	47.50	47.39	45.14	49.30	25.74	20.59	41.07	50.00	100.00
	Current	48.40	48.57	47.40	49.24	48.57	46.95	46.28	61.54	25.27	21.21	41.82	50.00	100.00
Mean number of foals per stallion, n	Historical	4.97±26.62	13.98±22.40	9.44±17.52	15.59±23.00	14.89±15.42	12.64±22.49	19.97±29.32	2.80±3.49	10.56±17.90	11.00±0.00	N/A	N/A	N/A
	Current	9.39±14.25	13.34±21.01	7.87±14.46	15.30±22.99	4.33±5.77	10.83±19.27	19.05±28.61	2.80±3.49	13.29±19.70	11.00±0.00	N/A	N/A	N/A
Maximum foal number per stallion, n	Historical	1804±26.62	366±22.40	219±17.52	272±23.00	40±15.42	65±22.49	128±29.32	9±3.49	57±17.90	11.00±0.00	N/A	N/A	N/A
	Current	236±14.25	366±21.01	219±14.46	272±22.99	11±5.77	50±19.27	128±28.61	9±3.49	57±19.70	11.00±0.00	N/A	N/A	N/A
Mares %	Historical	52.19	52.18	53.83	51.73	52.50	52.61	54.86	50.70	74.26	79.41	58.93	50.00	0.00
	Current	51.6	51.43	52.60	50.76	51.43	53.05	53.72	38.46	74.73	78.79	58.18	50.00	0.00
Mean number of foals per mare, n	Historical	4.30±3.42	4.00±3.27	3.53±3.05	2.91±2.57	2.75±1.92	3.23±3.42	2.74±2.14	2.23±1.48	2.56±2.14	1.50±0.54	1.20±0.45	2.00±0.00	N/A
	Current	3.70±2.75	3.57±2.71	2.97±2.44	2.62±2.05	2.50±2.07	1.54±0.97	2.47±1.75	3.00±2.65	2.02±1.41	1.57±0.54	1.20±0.45	2.00±0.00	N/A
Maximum foal number per mare, n	Historical	24.00±3.42	21.00±3.27	19.00±3.05	18.00±2.57	8.00±1.92	11.00±3.42	11.00±2.14	6.00±1.48	10.00±2.14	2.00±0.54	2.00±0.45	2.00±0.00	N/A
	Current	18.00±2.75	16.00±2.71	17.00±2.44	14.00±2.05	6.00±2.07	4.00±0.97	7.00±1.75	6.00±2.65	7.00±1.41	2.00±0.45	2.00±0.00	1.00±0.00	N/A
Stallion/Mare Ratio	Historical	0.92/1	0.92/1	0.86/1	0.93/1	0.90/1	0.90/1	0.82/1	0.97/1	0.35/1	0.26/1	0.70/1	1/1	0
	Current	0.94/1	0.94/1	0.90/1	0.97/1	0.94/1	0.89/1	0.86/1	1.60/1	0.34/1	0.27/1	0.72/1	1/1	0
Progeny from stallions selected for breeding, %	Historical	15.32	17.14	21.78	17.60	25.64	9.09	12.62	16.67	18.87	0	17.65	0	0
	Current	11.07	41.59	18.12	16.53	22.22	6.93	12.67	0	17.02	0	8.33	0	50.00
Progeny from mares selected for breeding, %	Historical	48.14	41.59	43.28	29.36	48.84	17.42	18.96	37.84	35.10	32.14	25.00	22.22	0
	Current	43.97	38.30	35.89	27.06	36.84	12.28	15.52	25.00	33.58	29.63	18.18	22.22	0

N/A: No offspring was registered.
